# Antimicrobial Effects on Swine Gastrointestinal Microbiota and Their Accompanying Antibiotic Resistome

**DOI:** 10.3389/fmicb.2019.01035

**Published:** 2019-05-15

**Authors:** Mohamed Zeineldin, Brian Aldridge, James Lowe

**Affiliations:** ^1^Integrated Food Animal Management Systems, Department of Veterinary Clinical Medicine, College of Veterinary Medicine, University of Illinois at Urbana-Champaign, Champaign, IL, United States; ^2^Department of Animal Medicine, College of Veterinary Medicine, Benha University, Benha, Egypt

**Keywords:** antimicrobial, gastrointestinal, microbiota, swine, resistome

## Abstract

Antimicrobials are the most commonly prescribed drugs in the swine industry. While antimicrobials are an effective treatment for serious bacterial infections, their use has been associated with major adverse effects on health. It has been shown that antimicrobials have substantial direct and indirect impacts on the swine gastrointestinal (GI) microbiota and their accompanying antimicrobial resistome. Antimicrobials have also been associated with a significant public health concern through selection of resistant opportunistic pathogens and increased emergence of antimicrobial resistance genes (ARGs). Since the mutualistic microbiota play a crucial role in host immune regulation and in providing colonization resistance against potential pathogens, the detrimental impacts of antimicrobial treatment on the microbiota structure and its metabolic activity may lead to further health complications later in life. In this review, we present an overview of antimicrobial use in the swine industry and their role in the emergence of antimicrobial resistance. Additionally, we review our current understanding of GI microbiota and their role in swine health. Finally, we investigate the effects of antimicrobial administration on the swine GI microbiota and their accompanying antibiotic resistome. The presented data is crucial for the development of robust non-antibiotic alternative strategies to restore the GI microbiota functionality and guarantee effective continued use of antimicrobials in the livestock production system.

## Introduction

**Figure 1:**
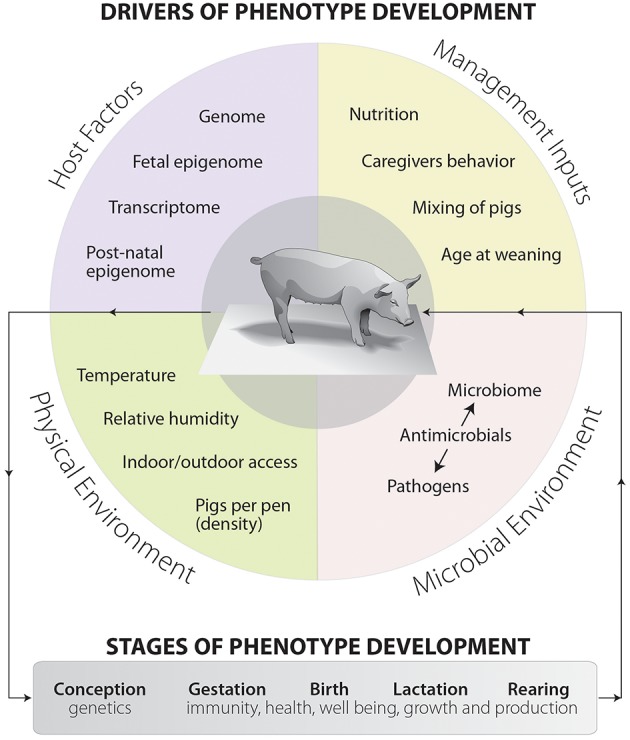
The drivers and main components of phenotype development in swine production systems. Phenotype development is made up of four separate but often blended sets of factors and overlapping components. These components are host factors, management inputs, microbial environment, and physical environment. The dashed arrows represent interactions between drivers and can occur between any drivers. This diagram also shows the stages of phenotype development, starting from conception and moving toward rearing.

Recently, the swine industry has focused on sustainable pork production which maximizes value over production costs and represents a shift away from antimicrobial usage. There is an urgent need not only for higher production efficiency to meet consumer expectations, but also for the development of new phenotypes related to host vitality and robustness (Merks et al., 2012). Phenotypic development in swine is a complex multistage process, starting from conception stage and continuing throughout the entire production cycle (Pluske, 2016). There are four major criteria that drive the phenotypic development and ultimately impact swine health, including host factors, management inputs, stable microbial ecosystem, and surrounding physical environment (Figure 1). Some human data and animal experiments have revealed that the crosstalk and interaction between microbial environment and other phenotypic drivers are the key distinguishers of host health (Blaut and Clavel, 2007; Metzler and Mosenthin, 2008). The swine microbial ecosystem is composed of rich and diverse populations that harbor thousands of different microbial species (aerobic, facultative anaerobic, and strictly anaerobic), dwelling in different anatomical biogeographic locations (Metzler and Mosenthin, 2008; Holman et al., 2017). These mutualist populations have a wide range of functions, including providing colonization resistance against potential pathogens, absorbing different kind of nutrients, modulation of the host's immune system, metabolizing indigestible polysaccharides, and regulating the host's metabolism (Bischoff, 2011; Venable et al., 2016). Therefore, alteration of the swine microbial environment may detrimentally influence the host's health status and inhibit the pathogens colonization (Marchesi et al., 2016). Understanding the mechanistic pathways and abundance of these alternations are required to discover new and different management practices to promote growth rate, increase efficiency of feed utilization, and improve overall swine health.

**Figure 2:**
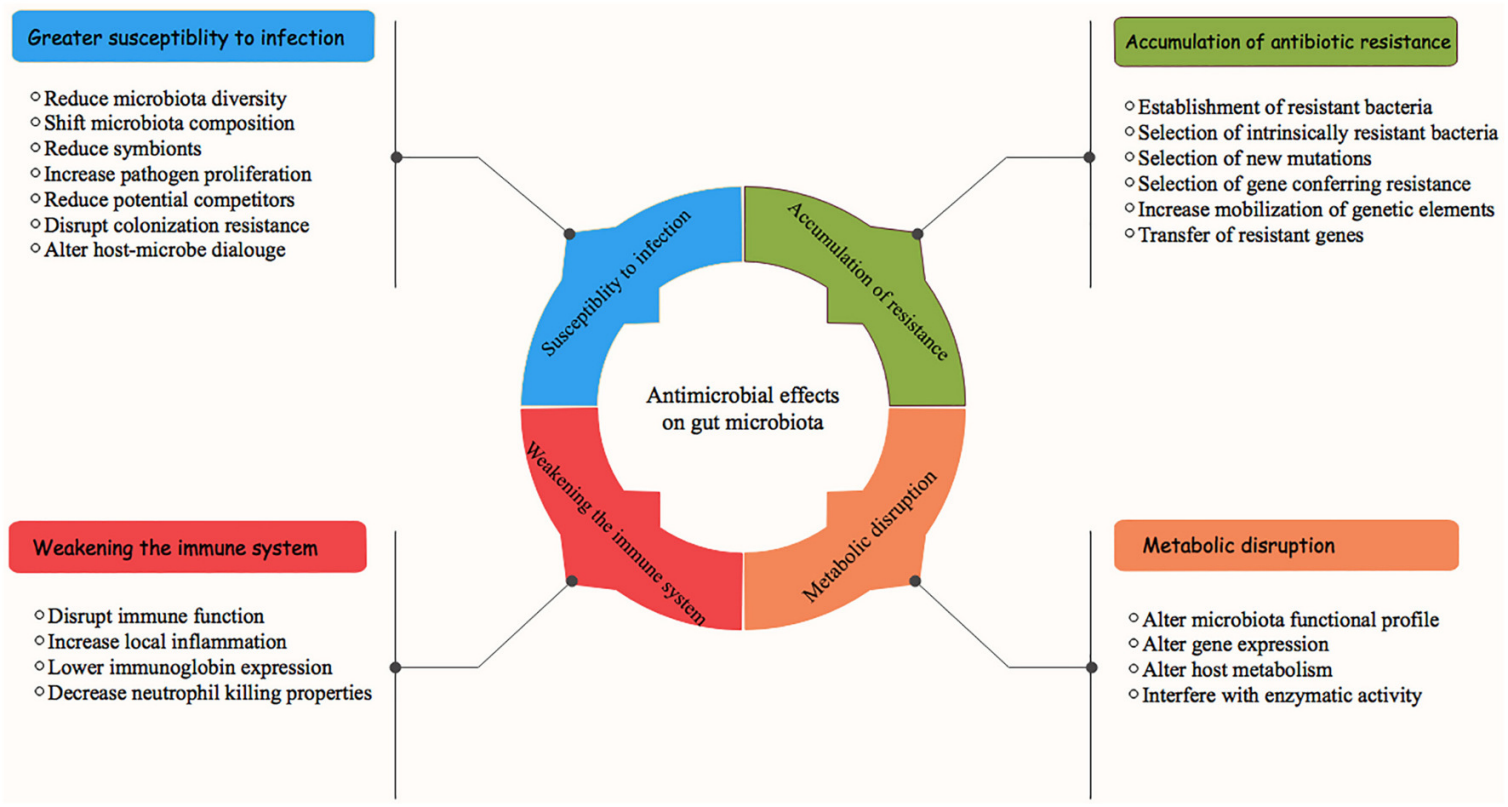
Antimicrobial effects on swine gastrointestinal microbiota and associated health consequences.

With recent advances in our understanding of swine microbial ecosystem structures and functions, we are becoming increasingly aware of the impacts of antimicrobial on mucosal microbiota and how its use negatively impacts the host's health (Zeineldin et al., 2018b). Equally important is the potential enrichment of antimicrobial resistome between the commensal microbiota as a result of antimicrobial use, which is one of the most vital public health issues that we currently face (Wright, 2007). The detrimental impacts of antimicrobial on the GI microbiota and host health are summarized in Figure 2. Traditionally, the impacts of antimicrobial administration on GI microbiota structures and development of antimicrobial resistance were largely characterized by culture-based techniques and/or a PCR-based approach, both of which underestimate the presence of novel ARGs (Zhu et al., 2013). Consequently, culture independent platforms (real-time PCR quantification, next generation sequencing, and functional metagenomics) have been used to efficiently quantify and assess the resistant opportunistic pathogens and emergence of antimicrobial resistome (Gerzova et al., 2015). While antimicrobial intervention disrupts GI microbiota structures and function, we are just beginning to estimate the relative contribution of its use on emergence of the antimicrobial resistome.

In this review, we present an overview of antimicrobial use in the swine industry and its association with the emergence of antibiotic resistance genes (ARGs). Additionally, we review our current understanding of GI microbiota and its role in swine health. Finally, we explore the effects of antimicrobial use on the swine GI microbiota and their accompanying antimicrobial resistome. The presented data is vital for the development of robust non-antibiotic alternative strategies to restore the GI microbiota functionality and guarantee effective continued use of antimicrobial in the livestock production system.

## Antimicrobial Use in Swine Management System

In the swine industry, antimicrobial has four potential uses: (1) disease treatment, (2) disease control, (3) disease prevention, and (4) increased the growth performance (O'Neill, 2014). It is therefore unsurprising that antimicrobial is the most commonly prescribed drug in the swine industry (Dumas et al., 2016). It is estimated that all food-producing animals consume more than 70% of antimicrobial produced worldwide. The pigs are usually raised in groups, in close proximity to one another. Many production systems use all-in, all-out management to control and prevent infectious disease outbreaks (Dewey et al., 1999). However, high contact rates provide optimal conditions for the spread of infectious diseases, many of which require the use of antimicrobials to minimize economic losses and welfare concerns. Estimates range from 62% of nursery production units and 44% of grower/finisher units (McEwen and Fedorka-Cray, 2002) to 33% of nursery units and 30% of grower/finisher units use of antimicrobial for growth promotion (Holman and Chénier, 2015). Data collected in 2001 by the USDA for US herds found that 70% used antimicrobials in starter feeds, 59% used them in grower/finisher feeds, and 46% used them in sow feeds (Cromwell, 2002), which were higher than the estimates of McEwen and Fedorka-Cray in similar populations (McEwen and Fedorka-Cray, 2002). According to the Food and Drug Administration (FDA), the available antimicrobial classes and chemotherapeutic agents (chemically synthesized agents with antimicrobial activity) for use in swine are listed in Table 1 (FDA, 2017). Certain classes of these antimicrobial are approved and validated for their ability to be successfully combined with other antimicrobials (e.g., chlortetracycline, penicillin, and sulfamethazine), whereas others cannot be combined with other antimicrobials.

**Table 1:** Available antimicrobial classes and chemotherapeutics agents for use in swine.

**Antimicrobial class**	**Antimicrobial drug**	**Spectrum and mode of action**	**Importance to human**
Aminocoumarins	- Novobiocin	Narrow-spectrum antimicrobial that may be bacteriostatic or bactericidal at higher concentrations, that act by inhibiting bacterial DNA gyrase and work by targeting the GyrB subunit of the enzyme involved in energy transduction	Not medically important
Aminoglycosides	- Dihydrostreptomycin - Gentamicin - Neomycin - Spectinomycin	Broad-spectrum and potent bactericidal antimicrobials that act by inhibiting bacterial protein synthesis	Medically important
Amphenicols	- Florfenicol	Broad spectrum, bacteriostatic antimicrobial that acts by binding to the 50S ribosomal subunit of susceptible bacteria, preventing bacterial protein synthesis. It may be bactericidal against some very susceptible organisms	Medically important
Cephalosporins	- Ceftiofur hydrochloride - Cephapirin - Ceftiofur crystalline free acid	Broad-spectrum, bactericidal antimicrobials that act by disrupting the synthesis of the peptidoglycan layer forming the bacterial cell wall	Medically important
Diaminopyrimidines	- Ormetoprim	Broad-spectrum, bacteriostatic antimicrobial that acts by mimicking the substrate of respective enzymes and inhibiting the enzyme by blocking the active site of the enzyme	Medically important
Fluoroquinolones	- Danofloxacin - Enrofloxacin	Broad-spectrum, bactericidal antimicrobials that act by inhibiting DNA synthesis	Medically important
Ionophores	- Lasalocid - Monensin - Narasin - Salinomycin	Broad-spectrum biologically active molecules produced by microorganisms (mainly spore-forming bacteria) that act by specifically increasing the ion permeability of the cell membrane	Not medically important
Lincosamide	- Lincomycin - Pirlimycin	Broad-spectrum and bacteriostatic antimicrobials that act by interfering with the synthesis of proteins	Medically important
Macrolides	- Erythromycin - Gamithromycin - Tilmicosin - Tulathromycin - Tylosin	Broad-spectrum antimicrobials, dependent on concentration and bacterial species, that are either bactericidal or bacteriostatic; which act by inhibiting protein synthesis	Medically important
Penicillins	- Amoxicillin - Ampicillin - Cloxacillin - Penicillin	Narrow-spectrum bactericidal antimicrobials that act by specifically inhibiting the transpeptidase enzyme that catalyzes the final step in cell wall biosynthesis, the cross-linking of peptidoglycan.	Medically important
Polymyxins	- Polymyxin B (colistin)	Narrow-spectrum bactericidal antimicrobial that acts by disruption of both the outer and inner membranes of bacteria.	Medically important
Polypeptides	- Bacitracin	Narrow-spectrum, bacteriostatic antimicrobial; may be bactericidal, depending on the antimicrobial concentration and the susceptibility of the specific organism. Bacitracin acts by inhibition of the incorporation of amino acids and nucleotides into the cell wall	Not medically important
Quinoxalines	- Carbadox	Bactericidal synthetic antimicrobial that is primarily effective against gram-positive bacteria, with little efficacy against some gram-negative bacteria. The mechanism of carbadox action is not known	Not medically important
Streptogramins	- Virginiamycin	Bacteriostatic antimicrobial that acts by inhibition of cell growth of gram-positive bacteria and by inhibition of protein synthesis in gram-negative bacteria	Medically important
Sulfonamides	- Sulfadimethoxine - Sulfamethazine	Broad-spectrum, bacteriostatic antimicrobials that act by interfering with folic acid synthesis by preventing addition of para-aminobenzoic acid into the folic acid molecule through competing for the enzyme dihydropteroate synthase	Medically important
Tetracyclines	- Oxytetracycline - Chlortetracycline	Broad-spectrum, bacteriostatic antimicrobials that act by inhibition of bacterial protein synthesis	Medically important

The antimicrobial spectrum, administration dosage, pharmacokinetics and pharmacodynamics vary greatly according to different antimicrobial classes and their chemical structures (Cromwell, 2002). Some antimicrobials are easily absorbed after both therapeutic and subtherapeutic administration (e.g., oxytetracyclines and sulfonamides), whereas other antimicrobials are poorly absorbed after administration (e.g., bacitracin). In swine industry, the duration of antimicrobial administration typically ranges from 20 to 40 days for disease prevention and control (Stone et al., 2009). Alternatively, for growth promotion, antimicrobials are generally used for a long period of time at relatively low concentrations. While the mode of action of antimicrobial growth promotion remains poorly characterized, several potential mechanisms have been proposed. These mechanisms include decreased production of harmful metabolites (metabolic effect), increased absorption of available dietary nutrients (nutritional effect), and reduction of endemic subclinical diseases (disease control effect; Dibner and Richards, 2005). It is remarkable that antimicrobial use as a growth promoter in younger pigs is consistently efficacious while little to no response is seen in older animals (Cromwell, 2002; Skinner et al., 2014). In growing piglets, the average duration of antimicrobial use for growth promotion ranges from 22.7 to 76.8 days (Dewey et al., 1997). This prolonged exposure to sub-therapeutic antimicrobial concentrations provides ample opportunity for antimicrobial resistance to develop, particularly when compared to therapeutic use (Aarestrup et al., 2008). Consequently, there is increasing consumer desire to make sub-therapeutic antimicrobial use less frequent in livestock production (Sommer et al., 2017).

Several published studies have addressed the safety of antimicrobials, all of which could not identify a direct link between antimicrobial use in livestock and human health (Phillips et al., 2004; Chang et al., 2015). In contrast, a systematic review about restricting antibiotic use in animals and its association with antibiotic resistance in human beings concluded that antimicrobial use in food-producing animals is recognized as one of the major contributors to development of resistant organisms that result in life-threatening human infections (Landers et al., 2013). But, in general, it seems inevitable that antimicrobial administration in animals and its relationship to human health remain unquantified.

## Association Between Antimicrobial Use and Antimicrobial Resistance

Since the discovery of antimicrobials, the main goal of its use in the swine industry has been to eliminate pathogenic microbes, thereby facilitating growth and restoration of beneficial microbial communities (Holman and Chénier, 2015). However, these goals are routinely complicated by presence and dissemination of ARGs among microbes (McEwen and Fedorka-Cray, 2002). Resistance to antimicrobials is a natural occurrence, developed by the microbes to help in their survival against other antibiotic-producing microorganisms in the surrounding environment (Phillips et al., 2004). In many cases, detection of clinical signs for a disease in an individual animal provokes prophylactic treatment for the whole herd (Founou et al., 2016). This approach can increase abundance of resistant bacterial strains and elevate the expression of ARGs (Langdon et al., 2016).

Traditionally, the impacts of antimicrobial treatment on emergence of antimicrobial-resistant bacteria have focused only on pathogenic bacteria (e.g., *Salmonella, E. coli, Shigella*, and *Enterobacter*; Founou et al., 2016). Many researches have investigated the association between antimicrobial use in livestock and development of antimicrobial resistance across the resident microbiota (Everaert et al., 2017; Johnson et al., 2017). When an antimicrobial is administered, it eliminates the susceptible microbial populations, leaving behind resistant strains that continue to evolve and multiply in its number (Founou et al., 2016). Selective pressure from antimicrobial exposure is exploited by antimicrobial-resistant microbes, providing them with an evolutionary advantage (Brandl et al., 2008). The resistant microbes, in presence of antimicrobials, also have a competitive advantage which facilitates its spread among other microbial populations in the surrounding ecosystem (Holmes et al., 2016). The dissemination of ARGs requires acquisition or transfer of genetic elements encoding antimicrobial resistance between the bacterial strains. The resistant bacterial populations transmit their genetic resistance pools to their progeny through vertical evolution or to other adherent bacterial species through horizontal transmission (D'Costa et al., 2007). Vertical gene transfer occurs during cell division, where resistant genes either on chromosomes or plasmids transfer to the progeny cells, leading to bacterial resistance (Lawrence, 2004). Alternately, horizontal gene transfer involves genetic pool exchange within and between the microbial populations, where genetic density and complexity of the commensal microbial community stimulate the spread of ARGs among microbes (Founou et al., 2016). The resistant genetic material is usually acquired by microbes either through conjugation, transformation, and/or transduction (Holmes et al., 2016). It is then possible for new mobile genetic element-associated transmission of antimicrobial resistance determinants to be incorporated into the bacterial chromosome or replicate independently (Sommer and Dantas, 2011). The presence of mobile genetic elements (plasmids, integrative conjugated elements, transposons, and integrons) are therefore important in transmission of antimicrobial resistance among microbes (D'Costa et al., 2007). The reservoirs of antimicrobial-resistant bacteria are ubiquitous and can merge with the GI resident microbiota through two different mechanisms (Holman and Chénier, 2015). First, the resistant bacteria can be acquired directly by the host and colonize the GI mucosal epithelium; secondly, a previously susceptible bacterial species can become resistant through induction of antibiotic-resistant mutants or through resistant gene transfer events (Crofts et al., 2017). While there is a clear association between the use of antimicrobial and emergence of antimicrobial resistance, this relationship is complex and influenced by multiple confounding factors (e.g., pathogen-host interactions, pathogen–drug interactions, rate of mutation, rate of transmission, cross-resistance, and co-selection of resistance to unrelated drugs; Holmes et al., 2016).

## Effect of Antimicrobial Intervention on Swine Gastrointestinal Microbiota

The term microbiome is widely used to describe the resident populations of different organisms (bacteria, viruses, fungi, archaea, and protists) that live and/or colonize the body of multicellular host and their genetic material (Turnbaugh et al., 2007). Swine GI microbiota is not uniform and differs drastically between individuals, even individuals raised in the same management system. Additionally, the relative abundance of specific bacteria differ according to different GI biogeographic locations (Leser et al., 2002; Maradiaga et al., 2018; Yeoman et al., 2018), with richer and more diverse communities in the colon compared to the ileum and stomach (Holman and Chénier, 2015). Understanding how GI microbiome composition affects swine health is an emerging area of research (Isaacson and Kim, 2012; Zeineldin et al., 2017a). However, the exact mechanisms of how GI microbiota contributes to swine health are still unclear. There are new studies endeavoring to increase our understanding about this mechanism (Pluske et al., 2018). GI mutualistic microbiota play an important function in bile salt recycling, volatile fatty acid production, cellulose digestion, metabolism of undigested carbohydrates, and nutrient recovery (Bischoff, 2011). Additionally, GI microbiota contribute to resistance against colonization of pathogenic microbes through competition for binding sites, nutrient utilization at mucosal epithelium, and modification of local environment (Mach et al., 2015). Therefore, understanding different factors that shape swine GI microbiota and their composition, particularly in early life, are required to discover new targets and/or develop novel management practices to promote optimal GI microbiota development.

With the advancement of methodologies to assess microbiota composition (Zeineldin et al., 2017b), several considerations have been raised regarding the impact of antimicrobial administration on the resident microbial populations in swine (Bokulich et al., 2016). There are several reports and longitudinal studies that attempt to understand the impacts of antimicrobial intervention on swine GI microbiota (Gerzova et al., 2015; Holman and Chénier, 2015; Oultram et al., 2015; Bokulich et al., 2016; Founou et al., 2016; Holman et al., 2018; Zeineldin et al., 2018a). Table 2 lists a summary of the existing metagenomic studies on the impacts of antimicrobial administration on swine GI microbial communities. Commonly, antimicrobial is given to wipe out pathogenic microbes during acute infection (Dewey et al., 1999). However, several antimicrobial classes are not specific, and consequently wipe out a wide range of resident GI microbiota that are beneficial and pivotal for health (Neuman et al., 2018). Recently, a comprehensive review by Langdon et al. revealed that short and long term antimicrobial intervention in humans drastically changes both adult and neonatal microbiota structure (Leibovitz et al., 2003; Langdon et al., 2016). This shift has been associated with an increased chance of subsequent GI disease (Pettigrew et al., 2012). Although the shifts in microbiota composition occurred after antimicrobial administration, some populations have returned to a pretreatment state within 4 weeks following a single-dose treatment. Other taxa, meanwhile, failed to return to pretreatment levels even after 6 months following treatment (Jernberg et al., 2010). Similarly, shifts in the GI microbiota in other animals after antimicrobial administration (a combination of metronidazole, amoxicillin and bismuth) dissipated after cessation of treatment (Schmidt et al., 2009). The precise components responsible for GI microbiota recovery after antimicrobial administration are still undefined. Recognition of different factors that promote microbiota recovery after antimicrobial administration open up new opportunities for development of novel therapies that promote the GI health.

**Table 2:** Summary of the existing metagenomic studies on the impacts of antimicrobial intervention on the swine gastrointestinal microbiota.

**Study population**	**Population age**	**Samples type**	**Sequencing platform and 16S rRNA gene hypervariable region**	**Antimicrobial class**	**Route of administration & duration**	**Data availability accession number**	**Finding**	**References**
20 piglets	8 weeks	Fecal swabs	Illumina MiSeq (V1–V3)	Ceftiofur crystalline free acid, ceftiofur hydrochloride, oxytetracycline, procaine penicillin G and tulathromycin	Parenteral-Single dose	PRJNA323521	Analysis revealed a pronounced, antimicrobial-dependent shift in the composition of fecal microbiota over time from day 0. By day 14, the fecal microbial compositions of the treatment groups had returned to a distribution that closely resembled that observed on day 0, but differences were still evident.	Zeineldin et al., 2018a
16 ileal-cannulated piglets	42 days	Fecal and ileal samples	Illumina MiSeq sequencing (V3–V4)	Ampicillin, gentamicin, and metronidazole	In feed for 13 days	SRP115601	Antibiotics administration increased abundance of Escherichia/ Shigella and decreased abundance of *Lactobacillus* and *Bifidobacterium*.	Gao et al., 2018b
30 piglets	Post-weaning	Feces	Illumina MiSeq sequencing (V1–V2)	Amoxicillin and colistin sulfate	In feed for 30 days	PRJNA445806	Antibiotics administration had a positive effect in the endogenous microbiota of post-weaning pigs with clear increase in abundance of *Bacillus* and *Lactobacillus* Spp.	Soler et al., 2018
16 litters of suckling piglets	7 days	Ileal and cecal digesta	Illumina MiSeq sequencing (V3–V4)	Olaquindox, oxytetracycline and kitasamycin	In feed from days 7 until days 23 of old	SRP 132384	Antibiotics administration significantly decreased bacterial diversity and richness in ileum. Antibiotics significantly reduced the abundance of *Lactobacillus* in both ileum and cecum, increased the abundance of *Streptococcus, Enterococcaceae, Fusobacteriales*, and *Corynebacterium* in ileum, and increased the abundance of *Ruminococcaceae* and *Erysipelotrichaceae* in cecum.	Yu et al., 2018
12 weaned piglets	45 days	Fresh digesta from the proximal ileum and feces	Illumina MiSeq sequencing (V3–V4)	Ampicillin, gentamicin, and metronidazole	Infused the antibiotic mixture with 10 mL saline through T-cannula in distal ileum for 25 days	SRP124814	The antibiotic infusion did not change the proximal ileal microbial composition, but it markedly altered the fecal microbial composition and increased aromatic amino acid metabolism.	Gao et al., 2018a
69 crossbred barrows	21 days	Intestinal contents from the ileum and colon	Illumina MiSeq sequencing (V3–V4)	Chlortetracycline and colistin sulfate	In feed for 28 days	SRP095386	In-feed antibiotic supplementation significantly increased *Spirochaetes, Tenericutes, Euryarchaeota, Verrucomicrobia, TM7* and reduced *Chlamydiae* in ileal digesta.	Yu et al., 2017
4 litters of crossbred piglets	After birth	The entire intestine was subsequently extracted.	Illumina MiSeq sequencing (V1–V3)	Amoxicillin	Oral feeding- twice daily at birth until day 14	None	Antibiotic administration exerted a transient impact on postnatal gut microbiota colonization and microbial metabolite production.	Li J. et al., 2017
60 weaning piglets	28-d-old	Luminal contents of the jejunum, ileum, cecum, and colon	Illumina MiSeq sequencing (V3–V4)	Colistin sulfate	In-feed-28 days	None	Antibiotic treatment resulted in modulation of gut microbiota with decreased abundance of *Acholeplasma, Arcobacter, Caldicoprobacter, Desulfotomaculum, Ignatzschineria, KSA1, Leptolyngbya, Natronincola_Anaerovirgula, Pseudomonas, Pseudoramibacter_Eubacterium, Tepidimicrobium*, and *Tissierella_Soehngenia*.	Li K. et al., 2017
187 piglets	7 days	Luminal content and scraping from stomach, duodenum, jejunum, ileum, cecum, and colon	Illumina MiSeq sequencing (V3–V4)	Olaquindox, oxytetracycline, kitasamycin	In feed from day 7 to 42	SRP102481	The early-life antibiotic intervention decreased the abundance of *Lactobacillus* in the stomach, increased the abundance of potentially pathogenic *Streptococcus suis* in the small intestine, increased the abundance of *Treponema* in the colonic lumen and increased abundance of *Faecalibacterium* in the ileal mucosa.	Mu et al., 2017
150 recently weaned pigs	25 days	Fecal samples	Illumina MiSeq sequencing (V4)	Bacitracin zinc, chlortetracycline and colistin	In feed for 28 days	None	Antibiotics caused shifts in microbiota composition and affected composition of gut microbiota in the different gut locations of the weaning piglets.	Li P. et al., 2017
9 weaned piglets	30 days	The contents of three intestinal segments (jejunum, colon, and cecum)	MiSeq sequencing (V3–V4)	Chlortetracycline	In feed for 10 days	SRP071319	Chlortetracycline treatment resulted in elimination of several taxa and increased abundance of *Firmicutes* and the genus *Prevotella*.	Zhang et al., 2016
All piglets of 16 sows	4 day	Jejunal content	Pig Intestinal Tract Chip (PITChip) version	Tulathromycin	Parenteral- single dose	Available upon request	Antibiotic treatment resulted in long-lasting effects on gut microbiota composition and host intestinal gene expression.	Schokker et al., 2015
6 piglets	3 weeks	Fresh fecal samples	Illumina MiSeq sequencing (V4)	Chlortetracycline, sulfathiazole, and penicillin (2:2:1)	In feed for 9 weeks	SRP045387	Antibiotics administration showed no growth-promoting effect but resulted in inhibition of the growth of potential pathogens.	Unno et al., 2015
6 piglets (3 male and 3 female)	3-19 weeks	Fecal swabs	Illumina MiSeq V4	Tylosin and Chlortetracycline	In feed for 70 days	SRP041290	Tylosin treatment resulted in a shift in the relative abundance of several taxa and in 26 operational taxonomic units, while chlortetracycline treatment resulted in minor alterations.	Holman and Chénier, 2014
12 piglets	3 months	Scraping and luminal content of ileum, cecum and mid-colon	Illumina MiSeq sequencing (V1–V3)	Chlortetracycline, sulfamethazine and penicillin (ASP250)	In feed for 3 months	PRJNA72355	ASP250 resulted in increased *Escherichia coli* populations in the ileum*, Lachnobacterium* spp. in all gut locations, and resistance genes to antibiotics not administered.	Looft et al., 2014a
6 piglets	3 weeks	Fecal samples	Illumina MiSeq sequencing (V1–V3)	Carbadox	In feed for 3 weeks	PRJNA237795	Carbadox treatment caused striking effects within 4 days of administration, with significant alterations in both community structure and bacterial membership, with increased *Prevotella* populations.	Looft et al., 2014b
24 pregnant sows	-	Feces	Roche 454 GS-FLX sequencer (V3–V5)	Mixture of: lincomycin, chlortetracycline, and amoxicillin.	In feed before delivery	None	Antimicrobial administration resulted in shifts in microbial community structure, increased proportion of resistant bacteria and genes.	Sun et al., 2014
4 pigs	28 days	Fecal material	Roche 454 GS-FLX sequencer (V1–V3)	Chlortetracycline	In feed for 4 weeks	None	No differences in alpha or beta diversity, nor at the taxa-level. Higher concentrations of chlortetracycline may be required to observe a shift in the gastrointestinal flora in swine feces compared with the low-level dose in this study.	Poole et al., 2013
10 pigs	10 weeks	Fecal samples	Roche 454 GS-FLX sequencer (V3)	Tylosin	In feed for 3 weeks	None	Tylosin administration caused shifts in both abundant and less abundant microbial species.	Kim et al., 2012
6 pigs	Recently weaned piglets	Freshly voided feces	Roche 454 GS-FLX sequencer (V1–V3)	ASP250	In feed for 18–21 days	None	ASP250 treatment resulted in a shift in bacterial phylotypes after 14 days of treatment, with the medicated pigs showing an increase in *Escherichia coli*, microbial functional genes related to energy production, and antibiotic-resistant genes.	Looft et al., 2012
12 piglets	3-7 weeks	Fecal samples	Roche 454 GS-FLX sequencer (V1–V3)	Carbadox and ASP250	In-feed- 1 weeks	PRJNA72355	ASP250, but not carbadox, caused significant population shifts in bacterial communities with non-significant changes in the abundance of antibiotic-resistant genes.	Allen et al., 2011

It is important, when quantifying the impacts of antimicrobial intervention on swine GI microbiota structure, to consider the ages of the studied populations, route of administration and the class of the administered antimicrobial (Neuman et al., 2018). While there are some similarities between the effects of antimicrobial administration on GI microbiota structure in growing and neonatal piglets, there are also significant dissimilarities due to distinct characteristics of the neonatal microbial composition. A recent study of 16 42-day-old ileal-cannulated pigs demonstrated that oral administration of ampicillin, gentamicin, and metronidazole treatment modified GI microbial population structure and function (Gao et al., 2018b). More precisely, use of ampicillin, gentamicin, and metronidazole decreased the *Lactobacillus* and *Bifidobacterium* abundance and increased the abundance of *Shigella* species by 256-fold compared to the control pigs (Gao et al., 2018b). Similarly, early life amoxicillin administration in neonatal piglets during the first 14 days of life exerted transient impacts on developing gut microbiota and decreased the genes involved in short-chain fatty acid signaling and pancreatic development (Li J. et al., 2017). In neonatal piglets, early life antimicrobial administration also resulted in differential dysbiosis of GI microbiota, with major alteration between different geographical locations. For instance, a mixture of olaquindox, kitasamycin, and oxytetracycline calcium administration decreased the relative abundance of beneficial *Lactobacillus* species and increased the relative abundance of potentially pathogenic *Streptococcus suis* in both the small intestine and stomach lumen (Mu et al., 2017). In growing piglets, antimicrobial administration also induced microbiota compositional changes in both abundant and less abundant GI microbiota. For example, tylosin-treated piglets showed higher relative abundance of *Lactobacillus, Eggerthella, Acetanaerobacterium*, and *Sporacetigenium* genera compared to control piglets (Kim et al., 2012). A mixture of amoxicillin and colistin sulfate treatment in post-weaning piglets also resulted in different digestive microbiota profiles along the entire gastrointestinal tract (Soler et al., 2018). Similarly, in-feed administration of colistin sulfate and bacitracin zinc in weaned piglets caused a significant shift in GI microbiota composition along different biogeographic gut locations (Li K. et al., 2017).

Published data also suggested that different classes of antimicrobial disrupt GI microbiota in different ways. This should be included in the decision-making process for antimicrobial prescription in livestock management systems. When assessing the impacts of in-feed sub-therapeutic concentrations of two common antimicrobials (tylosin and chlortetracycline) on swine GI microbiota composition, tylosin administration resulted in a major shift in the relative abundance of several taxa, while chlortetracycline administration only resulted in minor alterations (Holman and Chénier, 2014). Similarly, oral vancomycin and metronidazole have different effects on *Clostridium difficile*, where only vancomycin had an obvious impact on microbial community structure (Lewis et al., 2015). The simplest mechanistic explanation for variation in the swine GI microbiota response to antimicrobial intervention is due to differences in antimicrobial spectrum, route of administration, and degree of antimicrobial resistance (Kim et al., 2012; Looft et al., 2014a,b; Schokker et al., 2015; Mu et al., 2017; Soler et al., 2018).

## Gastrointestinal Microbiota as a Reservoir of Antimicrobial Resistome

The concept of the antimicrobial resistome was proposed by Gerard Wright in 2007 as a means of describing the collection of all known ARGs in the microbial ecosystem and their precursors at multiple levels (e.g., environment, pathogenic, and non-pathogenic microbes; Wright, 2007). Historically, determination of ARGs have primarily relied on conventional culture-based methods, with a focus on major pathogens that are readily cultured (Isaacson and Kim, 2012). While beneficial, these protocols do not provide information on the total amount of ARGs in the bacterial community as most species in that community cannot be cultivated, likely underestimating the complexity of the antimicrobial resistome (Henriksson et al., 1995). Although the antimicrobial resistome is theoretically accessible to all bacteria, the GI microbiota harbor a distinct antimicrobial resistome (Sundin and Wang, 2018). The known ARGs are likely to represent just a small portion of actual antimicrobial resistome populations. It is reasonable to assume that with the explosion of bacterial genome sequencing and functional metagenomics, many novel ARGs that were previously of unknown function and unrecognizable by sequence alone will be identified (D'Costa et al., 2007). The generation of more information about ARGs will be helpful in understanding the relationship between the resident microbial communities and their accompanying resistome (Boolchandani et al., 2019).

In parallel with the consecutive development of GI microbiota, the antimicrobial resistome is established during first few days of life or perhaps during prenatal phase even without prior exposure to antimicrobial treatment (Wright, 2007; Zeineldin et al., 2019). This concept endorses the theory that resistant bacteria and their antimicrobial resistome are established shortly after birth and are acquired either directly from the mother or through direct contact with resistant bacteria in surrounding environment (Gonzales-Marin et al., 2012). The GI microbiota has a large and diverse genetic pool that facilitates transmission of resistance between and within the resident commensal species (Sengupta et al., 2013). The effects of different antimicrobial intervention on emergence of the antimicrobial resistome has been extensively demonstrated (Wright, 2007; Enwemeka, 2013). In people, when the infants received antimicrobial treatment in the first 3 years of life, the GI microbiota expressed high levels of antimicrobial resistance compared to the control (Yassour et al., 2016). Similarly, the abundance of 149 ARGs conferring resistance to different classes of antimicrobials were detected in the swine feces from production units that used different antimicrobials either orally or via intramuscular injection (Zhu et al., 2013). Emergence of antimicrobial resistance determinants in pigs without prior antimicrobial administration has been also demonstrated previously (Pakpour et al., 2012; Agga et al., 2015), with the largest resistance category being against tetracycline antibiotic (Chambers et al., 2015). For instance, several tetracycline resistance genes (e.g., tetO, tetW, tetM, tetX, and tetQ), and macrolide resistance genes (e.g., ermG, ermF, and ermB) were frequently identified in the swine facilities in the absence of antimicrobial exposure (Looft et al., 2012). Similarly, our recent study showed that the neonatal piglets displayed a high frequency of ARGs without prior exposure of antibiotics (Zeineldin et al., 2019). Emergence of these ARGs without direct exposure to a known antibiotic also reveals that the swine GI antimicrobial resistome may not be affected by a reduction in antimicrobial administration in the swine industry (Holman and Chénier, 2015).

## Antimicrobial Alternatives in Swine Industry

The current efforts to define the complex composition of GI microbiota and how that community responds to antimicrobial intervention would improve our ability to develop novel non-antibiotic strategies to prevent GI infection in food-producing animals, subsequently increasing animal productivity (Marchesi et al., 2016). Considering this information, different management strategies are required to reduce the deleterious consequences of antimicrobials, particularly when its administration is needed to control bacterial infections. Broad discussions of possible antimicrobial alternatives have been summarized in Table 3 and were mentioned elsewhere (Potter et al., 2008; Allen et al., 2013, 2014; Papatsiros, 2013; Czaplewski et al., 2016). In this section, we will only focus on bacteriophage therapy as an important and promising example of available antimicrobial alternatives in the swine industry.

**Table 3:** Currently available alternatives to antimicrobials in swine industry.

**Antimicrobial alternative**	**Advantages**	**Possible disadvantages**	**Mechanism of action**	**References**
Phage therapy	- Phages are self-replicating - Lack of cross-resistance - Potential for modification - Low inherent toxicity - Biofilm clearance - Single and low dose potential - Relatively low cost	- Can be discovered by the host's immune system as a potential invader and may therefore rapidly be eliminated from the systemic circulation - Pharmacokinetic characteristics of phages are barely known - Phage therapy is time-sensitive - Bacteria can develop resistance to phages by mutation	Targets bacteria	Pires et al., 2017
Lysins	- Can quickly kill susceptible strains with a wider antibacterial spectrum - Selective toward specific strains of bacteria - Not prone to resistance development	- High cost - Easily degraded and lose activities during use and storage - Poor efficacy against gram-negative bacteria	Targets bacteria	Love et al., 2018
Antibacterial vaccine	- Inexpensive in production - Stable in storage	- Lack of relevant protective antigens - Lack of safety due to potentially harmful components - Killed vaccines require the use of adjuvants, which limits the delivery options for the vaccines	Primes host's immune response	Hoelzer et al., 2018
Antimicrobial peptides	- Not prone to resistance development - Broad-spectrum and bactericidal activity	- High production cost - Potentially toxic to cells - Unstable during transportation - Easily hydrolyzed by proteases in the gut	Targets bacteria	Wang et al., 2016
Phytobiotics	- Nutritional effect - Easy availability	- High variability - Pharmacokinetic characteristics of most of plants are not well-known - High risk of toxicity	Targets bacteria and improves gut health	Mohammadi Gheisar and Kim, 2018
Inhibitors for bacterial quorum sensing	- Not prone to resistance development	- The majority of QSIs cannot be widely applied because of their toxicity to eukaryotic cells - Only narrow-spectrum activity - High chance of degradation	Targets bacteria	Cheng et al. et al., 2014
Probiotics	- Easy availability - Relatively cheap - Not prone to resistance development	- Lack of standards - Causes several potential problems (animal poisoning, allergies, and diarrhea) - Cannot withstand low pH and bile acids in gastrointestinal tract - Difficult to reach high sufficient number of viable cells to colonize in the intestine	Improves gut health	Collins and Gibson, 1999
Prebiotics	- Promote immune functions - Show anti-viral activity - Have no residue - Not prone to resistance development	- Cannot inhibit and kill pathogens - Feeding large quantity of prebiotics may cause bloating, diarrhea, and other adverse reactions	Improves gut health	Collins and Gibson, 1999

Bacteriophage (phage) therapy involves the use of bacterial viruses (phages) to attack specific bacterial species, or a narrow group of microbes, without harming the resident autochthonous microbial communities (Kutateladze and Adamia, 2010). Because of their ubiquity in all natural environments and commercial swine facilities, as well as their specific action against pathogens, phages have been suggested as a promising antimicrobial alternative for use in swine (Zhang et al., 2015). Recent studies based on high throughput next-generation sequencing approaches highlighted the importance of phages in microbial evolution and bacterial community control (Pratama and van Elsas, 2018). In addition to GI microbiota inhabitants, the GI tract harbors diverse phage communities that have a synergistic effect along with the resident microbial communities to maintain GI health (Allen et al., 2013). Subsequent research studies demonstrated that bacteriophages attacks bacteria by attaching to the cell wall and injecting their genetic material into bacterial cytoplasm with subsequent integration into the bacterial genome. Phage populations are extensively diverse and generally grouped according to their morphological properties and life cycle into temperate (lysogenic) or virulent (lytic) phages. Virulent bacteriophages are natural predators of their bacterial hosts, they replicate using the host machinery, and complete their lifecycle by lysis of the host cell (Calero-Cáceres et al., 2019). In contrast, temperate bacteriophages integrate into the host's chromosome and produce a stable genetic relationship with the host during the process of lysogeny without creating new phage particles (Zhang et al., 2015). Despite the growing evidence that supports the medical importance of virulent bacteriophages, their functional potential in swine is not yet well-defined.

In the swine industry, bacteriophage intervention strategies have been extensively used to control various *Salmonella* serovars, *E. coli O157:H7, enterotoxigenic E. coli*-induced diarrhea and *Campylobacter* species (Lee and Harris, 2001; Nisbet et al., 2010; Harvey et al., 2011; Hooton et al., 2011; Cha et al., 2012). These studies have shown that phages can be effectively utilized against these pathogens. Most recently, a phage cocktail was used to reduce *Salmonella typhimurium* in artificially-infected market-weight swine (Wall et al., 2010; Hooton et al., 2011). Similarly, phage treatment in weaned piglets challenged with *S. typhimurium* via oral gavage reduced fecal and cecal *Salmonella* populations in phage-treated piglets compared to control piglets (Nisbet et al., 2010). Several other experiments have evaluated the antimicrobial ability of phages against *E. coli* infections. Oral administration of a phage cocktail was capable of reducing morbidity and mortality in enterotoxigenic *E. coli*-challenged pigs, even when used at the onset of clinical signs (Atterbury, 2009). Smith and Huggins also investigated the efficacy of a mixture of two phages against an enteropathogenic strain of *E. coli* in neonatal pigs. The results of this work indicated that phages which targeted adherence pili were more effective in controlling porcine *E. coli* than phages that target other pili (Smith and Huggins, 2009). Phage therapy was also associated with increased prevalence of beneficial microbes (e.g., *Bifidobacterium* and *Lactobacillus*) and decreased relative abundance of coliforms and *Clostridium* species in post-weaning piglets (Hosseindoust et al., 2017).

Since their discovery in 1915, phages have been proven to be harmless to humans, animals and plants. Compared to antimicrobial, phages are highly effective in killing their target bacteria without harming the rest of the microbiota in the ecosystem. Additionally, phages are relatively cheap, self-replicating, easy to isolate, and have low inherent toxicity (Sillankorva et al., 2012). Despite these advantages, there are many technical limitations in the implementation of phage therapy for treatment of infectious diseases in human and animals (Allen et al., 2014). Commercially available phages have a limited microbial range, are unstable, sensitive to temperature, have a narrow range of hosts, require rapid administration after infection, and could be neutralized by the host's immune system (Papatsiros, 2013; Zhang et al., 2015). Similarly to antimicrobial resistance, recent studies suggest that bacteriophages play a crucial role in the acquisition and emergence of the antimicrobial resistome (Calero-Cáceres et al., 2019). Phage genomes can harbor several antimicrobial resistomes belonging to different antimicrobial classes. Phage-resistant strains are believed to be generally less virulent than the phage susceptible wild types, but the use of a number of different phages in combination (phage cocktails) against many serotypes will likely alleviate this problem (Kutateladze and Adamia, 2010; Harvey et al., 2011). Therefore, high-throughput next-generation sequencing and genetic engineering will be necessary to create a more reasonable phage to optimize impact and create the best alternative to antimicrobial treatment.

## Conclusion

The application of both high-throughput next-generation sequencing and functional metagenomics have clarified the effects of antimicrobial administration on commensal populations as well as on emergence of ARGs. There is, therefore, a great interest in understanding the origins, evolution and totality of antimicrobial resistance, not just in pathogenic microbes but also in whole resident microbial environment. The evidence that the commensal population harbors a previously underappreciated antimicrobial resistome should shift the paradigm of what judicious use of antimicrobials in livestock means. In addition, it raises exciting questions about the acquisition and transfer of antimicrobial resistance cross GI microbiota. A better understanding of the impacts of specific antimicrobial intervention strategies on GI microbiota and their accompanying antimicrobial resistome could open the door to the development of a novel therapeutic approach in swine production systems.

## Author Contributions

MZ wrote the manuscript. BA and JL revised it. All authors have approved the manuscript submission.

### Conflict of Interest Statement

The authors declare that the research was conducted in the absence of any commercial or financial relationships that could be construed as a potential conflict of interest.
